# *Aegle marvels* (L.) Correa Leaf Essential Oil and Its Phytoconstituents as an Anticancer and Anti-*Streptococcus mutans* Agent

**DOI:** 10.3390/antibiotics12050835

**Published:** 2023-04-30

**Authors:** Alhussain H. Aodah, Mohamed F. Balaha, Talha Jawaid, Mohammed Moizuddin Khan, Mohammad Javed Ansari, Aftab Alam

**Affiliations:** 1 Department of Pharmaceutics, College of Pharmacy, Prince Sattam Bin Abdulaziz University, Al-Kharj 11942, Saudi Arabia; a.aodah@psau.edu.sa (A.H.A.);; 2 Department of Clinical Pharmacy, College of Pharmacy, Prince Sattam Bin Abdulaziz University, Al-Kharj 11942, Saudi Arabia; mohamed.balaha@med.tanta.edu.eg; 3 Pharmacology Department, Faculty of Medicine, Tanta University, Tanta 31527, Egypt; 4 Department of Pharmacology, College of Medicine, Imam Mohammad Ibn Saud Islamic University (IMSIU), Riyadh 13317, Saudi Arabia; 5 Department of Basic Medical Sciences, College of Medicine, Dar Al Uloom University, Riyadh 13314, Saudi Arabia; 6 Department of Pharmacognosy, College of Pharmacy, Prince Sattam Bin Abdulaziz University, Al-Kharj 11942, Saudi Arabia

**Keywords:** *Aegle marmelos*, essential oil, anticancer, antioxidant, anti-*Streptococcus mutans*, molecular docking

## Abstract

*Aegle mamelons* (*A. marmelos*) or Indian Bael leaves possess anti-cancerous and antibacterial properties and are used in the traditional medicine system for the treatment of oral infections. In the present study, the essential oil of the leaves of *A. marmelos* was explored for its anticancer, antioxidant, and anti-cariogenic properties. The hydro-distilled oil of *A. marmelos* leaves was analyzed using gas chromatography coupled with mass spectrometry (GC-MS). Monoterpene limonene (63.71%) was found to have the highest percentage after trans-2-Hydroxy-1,8-cineole and p-Menth-2,8-dien-1-ol. The MTT [3-(4,5-dimethylthiazol-2-yl)-2,5-diphenyltetrazolium bromide] assay was used to investigate the anticancer activity of the extracted oil against human oral epidermal carcinoma (KB), and the results showed significantly higher (**** *p* < 0.0001) anticancer activity (45.89%) in the doxorubicin (47.87%) when compared to the normal control. The antioxidant activity of the essential oil was evaluated using methods of DPPH (2,2-diphenyl-1-picrylhydrazyl) and ABTS (2,2′-azino-bis (3-ethylbenzothiazoline-6-sulfonic acid)). The results showed a significant (*** *p* < 0.001) percentage of inhibition of DPPH-induced free radical (70.02 ± 1.6%) and ABTS-induced free radical (70.7 ± 1.32%) at 100 µg/mL with IC_50_, 72.51 and 67.33 µg/mL, respectively, comparatively lower than standard compound ascorbic acid. The results of the molecular docking study of the significant compound limonene with the receptors tyrosinase and tyrosine kinase 2 supported the in vitro antioxidant potential. The anti-cariogenic activity was evaluated against *Streptococcus mutans* (*S. mutans)*. Results showed a significant minimum inhibitor concentration of 0.25 mg/mL and the killing time was achieved at 3 to 6 h. The molecular-docking study showed that limonene inhibits the surface receptors of the *S. mutans* c-terminal domain and CviR protein. The study found that *A. marmelos* leaves have potential anti-carcinoma, antioxidant, and anti-cariogenic effects on human oral epidermal health, making them a valuable natural therapeutic agent for managing oral cancer and infections.

## 1. Introduction

*Aegle marmelos* (L.) (*A. marmelos)* Corrêa (Rutaceae), commonly known as Bael, is a subtropical species commonly found in the South Asia region (India, Nepal, Bangladesh, Pakistan, and Sri Lanka), Southeast Asia (Myanmar, Vietnam, Cambodia, Philippines, Java), South America, and Africa [1,2]. The *A. marmelos* species are deciduous trees, reaching up to 10 m or higher with large or medium sizes of several branches. Fruits with hard pericarps are initially grey-green in colour and turn yellowish or orange at the ripening stage [2]. The petioles of *A. marmelos* are long and glabrous, and the leaves are approximately 2.5 cm long, alternate, trifoliate, single or compound, and aromatic [3]. Roots, bark, leaves, fruits, and seeds are used in Ayurveda and folk medicine systems to treat several disease conditions. In follicular medicine, *A. marmelos* is used to treat malabsorption, rheumatism, neurological diseases, cardiovascular problems, blood sugar, edema, dysentery, dyspepsia, vomiting, hair growth problems, childbirth, acute bronchitis, eye infection, ulcer, and intestinal worms [4]. In scientific studies, the leaves of *A. marmelos* are evaluated for antimalarial, anti-inflammatory, antispasmodic, antiviral, antimicrobial, antidiabetic, antioxidant, hyperlipidemic, and anticancerous activities [5,6].

Free radicals (FR) are responsible for initiating oxidation that causes disease conditions in human beings. Furthermore, activated oxygen facilitates the development of reactive oxygen species (ROS) in the body, which damages cells and causes several health problems [7]. The use of the synthetic antioxidants BHA (butylated hydroxy anisole), BHT (butylated hydroxytoluene), and TBHQ (tertiary butylhydroquinone) has been restricted because of their toxic or carcinogenic properties [8]. Essential oils have antioxidant effects that could be useful in maintaining healthy oral conditions [9]. Free radicals produced in the oral cavity can induce periodontal and dental caries [10]. Essential oils of the plant have antioxidant constituents without toxic or carcinogenic properties and may be a valuable source to protect the oral cavity from several oral infections [11].

Essential oils, volatile aromatic compounds obtained from various plant sources through steam distillation or other extraction methods, have emerged as potential therapeutic agents for combating cancer. Cancer has become a leading cause of death globally, and researchers are exploring new avenues to tackle this deadly disease. The vast repertoire of studies that focus on the anticancer properties exhibited by essential oils is a testament to their immense potential in healthcare. One such study highlights how *Aegle marmelos* leaves possess remarkable anticancer activity when subjected to rigorous scientific scrutiny, thus making it an ideal candidate for further research into developing novel therapies for cancer treatment [12,13]. Worldwide, mortality related to oral cavity cancer increased to 1.40-fold during the past three decades [14]. Squamous cell carcinoma (SCC) is a human epithelial carcinoma cell (KB cell line) and is the most common oral cavity cancer. It is the most common cause of oral cancer, representing up to 80–90% of all malignant neoplasms of the oral cavity [15]. About 95% of all head and neck cancers are related to oral squamous cell carcinoma (SCC), and about 50% of its incidence has increased over the last decades [16]. In a previous study, several natural essential oils, such as *Artemisia capillaris*, *Solanum spirale*, *Roxb*, *Curcuma longa* and *Ocimum basilicum*, have been investigated against KB cell lines [17,18].

Essential oils are well known for antimicrobial activity and are commonly used in oral hygiene products such as toothpaste and mouthwash. Today, consumers demand high-quality natural origin products in global markets. Several bacteria and fungi reported in previous studies are involved in oral infections, such as dental caries and periodontitis, which are serious oral health problems in most developed countries [19,20]. Furthermore, several recent scientific works have suggested that oral infections are mostly associated with pneumonia, diabetes, rheumatoid arthritis, Alzheimer’s, and cardiovascular disease [21]. More than seven hundred microbial species colonize human oral cavities, and each individual carries hundreds of microbial species [22]. Several bacterial and fungal species together form biofilms on the surface of the teeth called dental plaques [23]. Resistance of pathogens to antimicrobial drugs may be one of the significant problems in the management of oral infection [24]. In previous studies, several essential oils from plants have been tested against oral bacteria, such as *Satureja khuzestanica*, *S. bachtiarica*, and *Zataria multiflora*, propolis, tea tree, peppermint, and thyme [25,26,27]. Several works have been published on the effect of essential oils and their isolated compounds on oral microorganisms [28,29]. The essential oil of *A. marmelos* also exhibits antibacterial effects against several Gram-positive and harmful bacteria [30].

Extensive research studies have revealed diverse pharmacologically active metabolites in the volatile composition of *A. marmelos* leaves, showing its immense therapeutic potential. These biologically significant compounds hold promising anti-inflammatory, antimicrobial, and antioxidant properties that may effectively combat several chronic disease conditions prevalent in human body. These phytochemicals’ multidimensional medicinal benefits make *A. marmelos* a vital natural resource for developing novel drugs and therapeutics to ease human suffering [31,32]. Upon analyzing the chemical composition of *A. marmelos* leaves, it has been discovered that they contain many volatile oils. These essential oils have proven vital for various purposes due to their unique properties and diverse range, making them an essential asset in many industries. Among the numerous types of compounds found in these oils, some prominent ones include limonene, α-phellandrene, β-Caryophyllene, α-Humulene, γ-Muurulene, (E)-β-ocimene, α-pinene, β-elemene, germacrene, etc., each with its distinctive fragrance profile and benefits. It is evident that intensive research conducted on *A. marmelos* leaves’ oil content has revealed remarkable information about its complexity and versatility as a plant extract source. As such, the findings offer new insights into potential applications for this natural resource across multiple fields, including pharmaceutical production or perfumery industry formulations [12,33,34].

The development and design of drugs is a critical area of scientific investigation, potentially yielding significant benefits for both industrial and therapeutic purposes. Through extensive research into various pharmacological compounds’ effects, experts can better understand their molecular structure and activity relationship (SAR). In recent years, scientists have increasingly turned to computer-aided drug design methods, such as molecular modelling, as powerful tools for exploring SAR. With this cutting-edge approach, researchers can conduct complex analyses that delve deeply into the underlying mechanisms governing how these compounds interact with biological systems. One up-and-coming technique in this field is molecular docking studies or MD. MD has emerged as one of today’s most competent tools in drug design and development work by utilizing sophisticated algorithms capable of analyzing vast amounts of data about candidate molecules. With insights gained through advanced techniques like these, scientists may unlock new pathways towards groundbreaking discoveries that could continually transform medicine [35,36].

The study on *A. marvels* (L.) Correa leaf essential oil and its phytoconstituents as an anticancer and anti-*Streptococcus mutans* agent has been motivated by the pressing need to find effective treatments against cancer and bacterial infections. The researchers behind this investigation understand the potential of natural compounds found in *A. marvels* (L.) Correa leaves, exploring their properties as a promising source for therapeutic agents with significant medicinal benefits. Their findings suggest that these plant-based extracts may hold great promise for combating both cancerous cells and harmful bacteria such as *Streptococcus mutans*, offering hope for better health outcomes among patients suffering from these conditions.

In light of the pressing need to explore natural sources for cancer treatment, the present study undertook a comprehensive investigation into the chemical compositions and potential therapeutic applications of essential oil extracted from *A. marmelos* leaves. Specifically, we aimed to not only examine its anticancerous effects on human oral epidermal carcinoma cell lines (KB), but also assess its in vitro antioxidant properties as well as anti-cariogenic activities against oral *S. mutans*. In addition to these experimental analyses, we took our investigations one step further by conducting molecular-docking studies that allowed us to predict how significant active metabolites present in *A. marmelos* essential oil may exert their antioxidant and anticarcinogenic mechanisms within cells.

Through this multi-faceted approach, our study provides a more nuanced understanding of how *A. marmelos* can be used towards developing novel therapies for various diseases such as cancer and cariogenic dental conditions—opening up new avenues for future therapeutic uses.

## 2. Results

### 2.1. Gas Chromatography-Mass Spectrometry (GC-MS) Analysis

**Figure 1:**
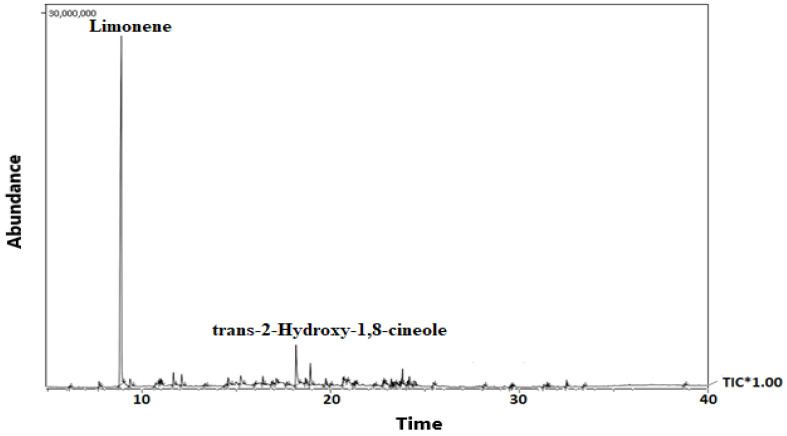
GC-MS chromatogram of *A. marmelos* leaf essential oil.

Essential oil from *A. marmelos* leaves produced 0.53% *v*/*w* of volatile compositions. The GC–MS chromatogram and the details of the volatile metabolites are shown in Figure 1 and Table 1, respectively. Limonene, a monoterpene (63.71%), was found to be the principal volatile component, along with the other monoterpenes trans-2-Hydroxy-1,8-cineole (6.85%), p-menth-2,8-dien-1-ol (2.29%) and menthyl acetate (2.02%). About 84.02% of the total volatile metabolites were monoterpenes, while the others were sesquiterpenes (6.01%), non-terpenes (8.01%), and Diterpene (0.28%).

### 2.2. Anti-Carcinoma Effects of A. marmelos Leaves Essential Oil

**Figure 2:**
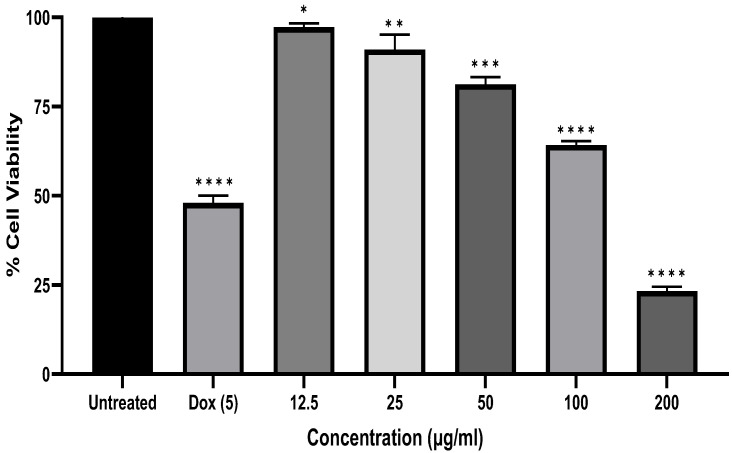
Effect of *A. marmelos* leaf essential oil on cell viability, where * *p* < 0.05, as compared with control; ** *p* < 0.01, *** *p* < 0.001 and **** *p* < 0.0001, as compared with control when treated with cancer cells at various concentrations (12.5 to 200 µg/mL).

Figure 2 shows the effects of *A. marmelos* leaf essential oil on cell viability at 12.5 µg/mL to 200 µg/mL by MTT assay in human epithelial carcinoma cells (KB cell line). The essential oil treatment significantly inhibited KB cell production after 24 h. In the present study, the IC_50_ value of essential oil was measured using the the linear regression equation y = mx + c, where y = 50 and m and c values were derived from the viability graph. The percentage of KB cells was significantly reduced by the oil dose-dependently, with an IC_50_ value of 132.52 µg/mL.

The MTT (3-(4,5-Dimethylthiazol-2-yl)-2,5-Diphenyltetrazolium Bromide) assay is a calorimetric assay for determining cytotoxicity and cell proliferation based on reducing tetrazolium dye MTT to formazan crystals. Mitochondrial lactate dehydrogenase (LDH) formed by living cells reduces MTT to insoluble formazan crystals (yellow colored). After dissolution in a suitable solvent, it showed a purple color; the intensity of the color is directly proportional to the number of viable cells. At 570 nm, it was measured spectrophotometrically. The outcomes of the statistical data of the cell cytotoxicity study by MTT demonstrated that *A. marmelos* leaf essential oil has significant cytotoxic potential against human epithelial carcinoma cells.

### 2.3. Antioxidant Activity of A. marmelos Leaves Essential Oil

**Figure 3:**
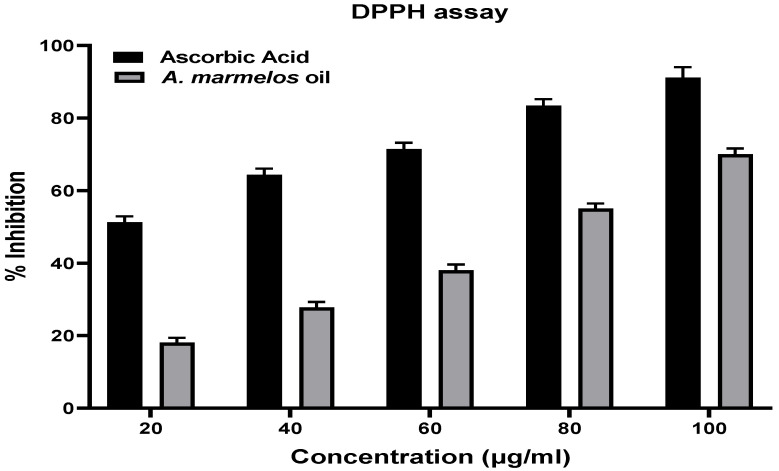

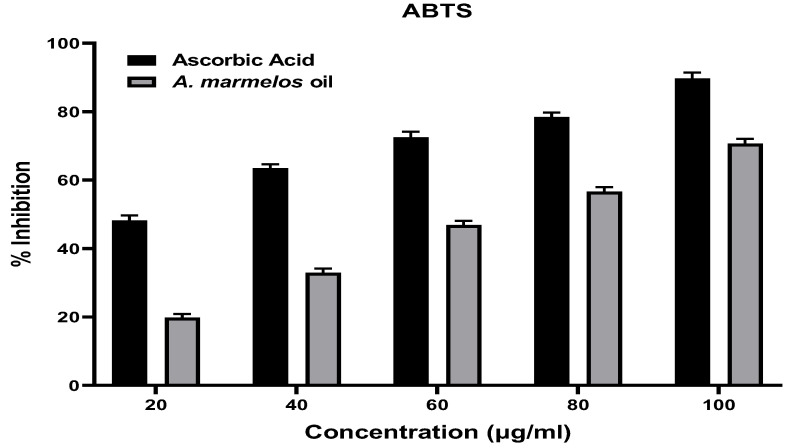
Percentage (%) inhibition of standard ascorbic acid and *A. marmelos* leaf essential oil on DPPH- and ABTS-induced free radical assay, where a significant *p*-value was determined when essential oil compared with standard ascorbic using GraphPad Prism 9.5.0, paired *t*-test.

To explore the antioxidant potential of *A. marmelos* leaf essential oil, the present study exhibited the DPPH and ABTS assays, and the outcomes of both free radical assays were reported in Figure 3. The percentage of inhibition of the DPPH-induced free radical capacity of *A. marmelos* leaf essential oil ranged from 18.05 ± 1.33 to 70.02 ± 1.6% at concentrations ranging from 20 µg/mL to 100 µg/mL. The range of inhibition of the ABTS-induced free radicals increased from 19.82 ± 1.05% to 70.7 ± 1.32% as the concentration increased from 20 µg/mL to 100 µg/mL. The inhibition of DPPH-induced free radicals by ascorbic acid ranged from 51.32 ± 1.55% to 91.11 ± 2.88% at concentrations ranging from 20 µg/mL to 100 µg/mL, while the range of inhibition of ABTS-induced free radicals increased from 48.27 ± 1.42% to 89.68 ± 1.72% as the concentration increased from 20 µg/mL to 100 µg/mL.

### 2.4. Anti-S. mutans Activity of A. marmelos Leaves Essential Oil

**Figure 4:**
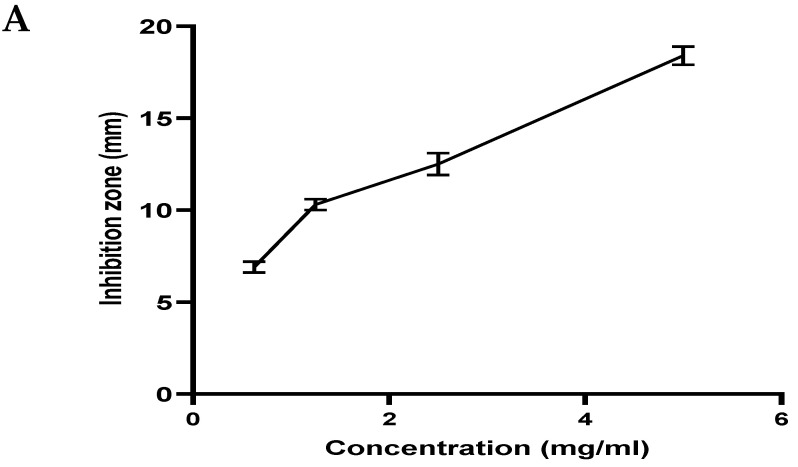

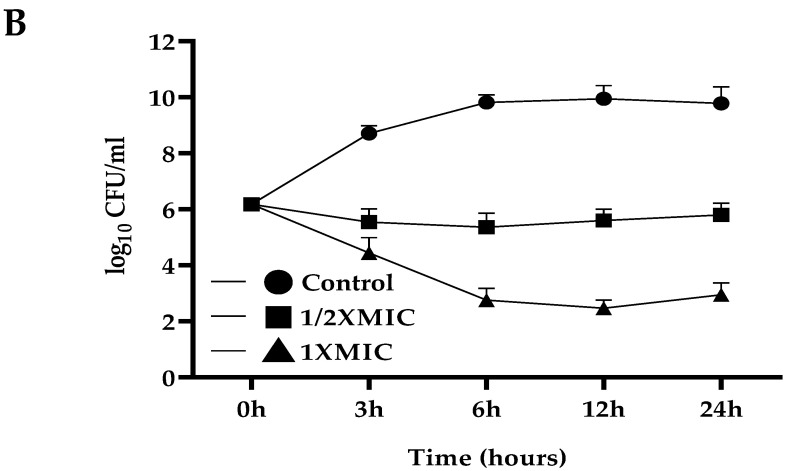
Zone (mm) of inhibition of (**A**) *A. marmelos* leaf essential against *S. mutans,* and time-kill assays of (**B**) *A. marmelos* leaf essential against *S. mutans*.

The agar diffusion assay explored the antibacterial activity of *A. marmelos* leaf essential oil. Figure 4A, shows the zone of inhibition of *A. marmelos* leaf essential oil against *S. mutans*. The zone of inhibition (ZOI) effect was observed from 0.625 mg/mL to 5 mg/mL against the *S. mutans* (18.4 ± 0.3 mm).

The cariogenic bacteria *S. mutans* exhibited a dose-dependent inhibition zone ranging from 6.9 to 18.48 mm, comparatively lower than the positive control, amoxicillin (at 10 µg/mL, inhibition zone 24.23 ± 0.8 mm). The MIC value of *A. marmelos* leaf essential oil against *S. mutans* was 0.25 mg/mL. The time-kill assay was performed using *A. marmelos* leaf essential oil against *S. mutans*, and the results are shown in Figure 4B. The outcomes of the control group (untreated *S. mutans*) exhibited approximately 6.5 to 9 (log10 CFU/mL) growth. In contrast, in the *A. marmelos* leaf essential oil 1/2 × MIC (0.125 mg/mL) treated group, the growth of *S. mutans* remained constant with 5.3 to 5.8 (log10 CFU/mL) growth. On the other hand, after the treatment with *A. marmelos* leaf essential oil 1 × MIC (0.25 mg/mL), the growth of *S. mutans* decreased drastically from 3 to 6 h, and it remained constant at approximately 2.7 to 2.59 (log10 CFU/mL) (Figure 4B). The plot deviated with 1 × MIC treatment inhibition of *S. mutans* growth, different from at 1/2 × MIC; hence, the results indicate that 1 × MIC of *A. marmelos* leaf essential oil showed a more rapid killing effect than 1/2 × MIC.

### 2.5. Molecular Docking Study of the Major Compound Limonene

Molecular docking studies were generally employed to assess the interaction between ligand–target proteins to explore the mechanism of action of the pharmacologically active compounds. The major volatile metabolite limonene was selected for the molecular docking study.

**Figure 5:**
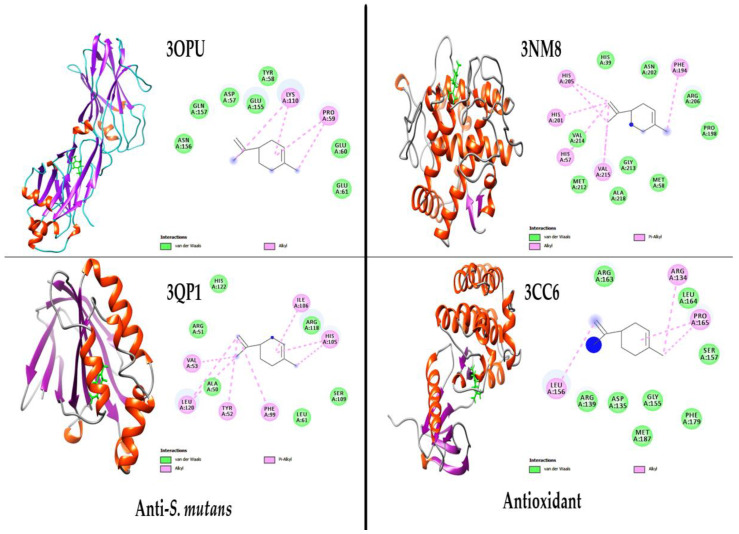
Interaction of ligand D-Limonene with antibacterial PDB-Protein (3OPU and 3QP1) and antioxidant PDB protein (3NM8 and 3CC6). The left of each figure shows a 3D surface pose (red sticks). The right figure shows the 2D pose where D-Limonene binds with different residues of enzymes.

Molecular docking was performed against antioxidant target enzymes [tyrosinase (PDB: 3NM8) and tyrosine kinase 2 beta, PTK2B (PDB: 3CC6)], and antibacterial [C-terminal domain of *S. mutans* surface protein (PDB: 3OPU) and CviR protein (3QP1)] receptors were used to explore critical ligand-protein interactions (Figure 5). Table 2 shows the results of energy ΔG (kcal/mol), inhibition constant (µM), and amino acid residues.

The complexes formed between the tyrosinase (3NM8) of antioxidant enzymes and limonene ligands exhibit stable chemical bonds with the active sites of the enzymes PHE (A194), VAL (A215), HIS (A57), HIS (A201), and HIS (A205), with a high binding energy (−7.73 kcal/mol) and inhibition constant (898.74 µM). Similarly, the complexes formed between tyrosine kinase 2 beta (PTK2B) (3CC6), and limonene exhibit stable chemical bonds with the active sites of the enzymes ARG (A134), PRO (A165), and LEU (A156), with a high binding energy (−7.40 kcal/mol) and inhibition constant (859.54 µM).

The complexes formed between the C-terminal domain of *Streptococcus mutans* surface protein SpaP (PDB: 3OPU) and of anti-*S. mutans* protein and limonene ligands exhibit stable chemical bonds with the active sites of the enzymes PRO (A59) and LYS (A110), with a high binding energy (−7.31 kcal/mol) and inhibition constant (987.29 µM). Similarly, the complexes formed between the protein CviR ligand-binding domain, bound to the native ligand C6-HSL (PDB: 3QP1), and limonene exhibit stable chemical bonds with the active sites of the enzymes ILE (A106), HIS (A105), PHE (A99), TYR (A52), LEU (A120), and VAL (A53) with a high binding energy (−7.89 kcal/mol) and inhibition constant (764.34 µM).

## 3. Discussion

The volatile compounds in the present sample exhibited limonene (63.71%) as the primary component, with trans-2-hydroxy-1,8-cineole (6.85%), p-menth-2,8-dien-1-ol (2.29%), and menthyl acetate as minor components. On the other hand, the composition of Egyptian essential oil compounds of *A. marmelos* leaves exhibited α-phellandrene (20.97%), α-pinene (17.76%) and δ-care (16.37%) as the major volatile components, different from the present sample [32]. Verma and coworkers (2014) reported that the chemical composition of limonene (31.0–90.3%), along with α-phellandrene (E)-β-ocimene, α-pinene in the essential oil from Indian *A. marmelos* exhibited similar but different quality from the present samples [34]. In the other study, Singh and coworkers (2009) reported the GC-MS analysis of *A. marmelos* leaf essential oil and reported limonene as the primary component [33]. Pant and coworkers (2019) worked on Nepali *A. marmelos* leaf essential oil and reported that sesquiterpenes, β-Caryophyllene (26%) is a significant compound, different from the present analysis of volatile metabolites [12]. Jamal and coworkers (2017) worked on Bangladeshi *A. marmelos* leaf essential oil and reported that ledene oxide-(II) (18.16%) is a significant compound, different from the present analysis of volatile metabolites [30]. Interestingly, Kaur and coworkers (2006) reported monoterpenes limonene as the major volatile component and the presence of total monoterpene (93.7%) and sesquiterpene (3.1%), supporting the present finding of major component limonene as well as the volatile content of total monoterpenes (83.2%) and sesquiterpene (5.5%) [37]. Several other studies also supported the present finding that limonene is the volatile primary component of the essential oil of *A. marmelos* leaves [33,38].

Several studies focused on the anticancer effects of volatile oils as natural bioactive sources that can inhibit cancer cells [39]. The cytotoxic effect of *A. marmelos* leaf essential oil on the KB cells was studied using an MTT assay. The outcome suggested that the induction of *A. marmelos* inhibited the viability of KB cells and leaves the essential oil in a dose-dependent manner. The value of the standard doxorubicin compound IC_50_ (2.55 µg/mL) has been reported for the KB cells in the prior study and comparatively showed a very high IC_50_ value compared to the essential oil of the present sample [40]. Lampronti and coworkers (2019) reviewed Bangladeshi *A. marmelos* and its antiproliferative effects. They reported that it might inhibit the proliferation of human leukemic-K562, B-lymphoid Raji, T-lymphoid-Jurkat, erythro-leukemic HEL, melanoma-Colo38, and breast cancer-MCF7 and MDA-MB-231 tumor cell lines [41]. Poonkodi and coworkers (2019) studied Western Ghats Region-South India *Aegle marmelos* (L.) Correa essential oil that showed significant antiproliferative effects against HeLa (human cervical cancer cell line) and less sensitivity against the African Monkey-Kidney normal cell line, Vero [13]. Several other studies also supported the anticancer effects of the essential oil of *A. marmelos* leaves [2].

Veerappan and coworkers (2007) reported that intraperitoneal (I.P) administration of *A. marmelos* leaf extract at doses of 50–100 mg/kg body wt. for 14 successive days to male and female Wistar rats did not show any toxicity [42]. Limonene is a major compound found in the essential oil of *A. marmelos* leaves and it has reported nontoxic, non-carcinogenic activity, and no compound-related clinical signs or histopathologic lesions were observed at doses up to 500 mg/kg body wt. in both rats and mice [43]. Hence, the essential oil extracted from the leaves of *A. marmelos* may have nontoxic effects with significant anticancerous potential, possibly due to the presence of the major metabolite limonene [44,45].

Both antioxidant methods involve the reduction of a colored oxidant that is produced due to an electron transfer, and it is easily monitored via UV. The DPPH assay is based on reducing DPPH (purple) radical to 1,1-diphenyl-2-picryl hydrazine. In contrast, ABTS involves the reduction of ABTS (blue/green) to a colorless sulfonic acid [46]. The ascorbic acid’s (standard) antioxidant potential was the highest in both methods. *A. marmelos* leaf essential oil presented a significant antioxidant potential, with IC_50_ values of 72.71 μg/mL for the DPPH assay and 67.33 μg/mL for the ABTS assay. The antioxidant activity of *A. marmelos* leaf essential oil exhibited dose-dependent scavenging activities in both assays, representing the significant antioxidant potential of *A. marmelos* leaf essential oil. Several studies have been conducted to determine the antioxidant properties of *A. marmelos* leaf essential oil [3,47]. The antioxidant activity may be due to limonene or other compounds in *A. marmelos* leaf essential oil [13,48].

*Aegle marmelos* can treat several conditions, including cancer, infections, and pain [5,6]. The present studies highlight the use of *Aegle marmelos* leaf essential oil in the treatment of oral cancer and dental infections. Both types of bacteria (Gram-positive and Gram-negative) are responsible for oral or dental infections. A scientific report suggested that Gram-positive bacteria from the genus Streptococcus are the most responsible [49]. *S. mutans* of the *Streptococcus* genus are most responsible for oral infections and dental caries [50]. The outcome of the present antimicrobial study of *A. marmelos* leaf essential oil showed significant inhibition against *S. mutans,* with MIC values of 0.25 mg/mL. Several scientific studies have shown the antimicrobial effects of *Aegle marmelos* leaf essential oil against Gram-positive and Gram-negative bacteria and fungi [33,38]. The antimicrobial effect of *Aegle marmelos* leaf essential oil are not restricted to *S. mutans* only, they also affect other types of oral microorganisms, such as *Bacillus cereus*, *S. aureus*, *Corynebacterium diphtheriae*, *Candida albicans*, *Bacillus subtilis*, *Escherichia coli* and *Candida albicans*, which are responsible for oral infections and dental caries [32,38]. The biological activities of *Aegle marmelos* leaf essential oil may be due to a high content of volatile metabolite limonene (63.71%) or several other metabolites such as trans-2-Hydroxy-1,8-cineole (6.85%), Methyl acetate (2.83%), p-Menth-2,8-dien-1-ol (2.29%) [51,52,53,54]. In the literature and review, several studies have reported that the natural monoterpene limonene has antioxidant and antibacterial activities [55].

The aim of this study was to explore the anticancerous, antioxidant, and antibacterial activity of *Aegle marmelos* leaf essential oil against oral disease conditions. Hence, a molecular docking study of limonene was simulated with tyrosinase (3NM8), tyrosine kinase 2 beta, PTK2B (3CC6), C-terminal domain of *Streptococcus mutans* surface protein SpaP (3OPU), and CviR ligand-binding domain bound to the native ligand C6-HSL (3QP1) targets proteins. The binding energy inhibition constant with several residues of H-bonding outcomes of enzyme tyrosinase with ligand limonene interaction supported the antioxidant properties of the *A. marmelos* leaf essential oil, mainly due to the presence of limonene. The inhibition of tyrosinase enzyme played a pivotal role in preventing melanin biosynthesis in the skin and hydroxylation of **L**-tyrosine to DOPA (3,4-dihydroxyphenylalanine) and further oxidation of DOPA to dopaquinone [56]. Therefore, tyrosinase inhibitors are an attractive target for evaluating antioxidant potential. Similarly, the binding energy inhibition constant with several residues of H-bonding outcomes of receptor tyrosine kinase 2 beta, PTK2B with ligand limonene interaction supported the antioxidant properties due to the presence of limonene. The antioxidant potential is mainly due to the inhibition of tyrosinase and tyrosine kinase 2 beta [57]. The antioxidant vitamin C has been reported previously to inhibit tyrosinase production [58].

To predict the anti-*S. mutans* mechanism of *Aegle marmelos* leaf essential oil, the major metabolite limonene was docked to the C-terminal domain of *Streptococcus mutans* surface protein SpaP. The results showed a high binding energy and inhibition constant with few H-bonding residues. The inhibition of the C-terminal domain of the *Streptococcus* mutant’s surface protein was essential in preventing dental caries [59]. Therefore, *Streptococcus mutans* surface protein inhibitor limonene may be an attractive ligand for anti-*S. mutans* or anticaries potential. Similarly, the binding energy and inhibition constant, with several residues of the H-bonding outcome of receptor CviR ligand-binding domain, bound to the native ligand C6-HSL with several residues conferring H-bonding stability to the complex, were explored with ligand limonene interaction and supported the antibacterial potential of limonene. The previous study suggested that the antibacterial potential of limonene is mainly due to the inhibition of the CviR ligand-binding receptor [60].

## 4. Materials and Methods

### 4.1. Chemicals

The cell line (oral adenocarcinoma cell line KB—human) was obtained from the National Center for Cell Science (NCCS, Pune, India), along with Dulbecco’s modified Eagle’s medium (DMEM), high glucose medium (Cat No: AL007, HiMedia, (Mumbai, Maharashtra, India), MTT reagent (HiMedia), fetal bovine serum (FBS), and Dulbecco’s phosphate buffered saline (D-PBS, Himedia). Dimethyl sulfoxide (DMSO), doxorubicin, ascorbic acid, and amoxicillin were purchased from Sigma-Aldrich (Mumbai, India). All media used for the antimicrobial studies were purchased from HiMedia Laboratories (Mumbai, India). 96-well plates for cell culture (Kennebunk, ME, USA) and all other chemicals (Analytical grade) used in the present study were purchased from HiMedia and Sigma Aldrich (Mumbai, India).

### 4.2. Methods

#### 4.2.1. Extraction of Essential Oil

*Aegle marmelos* (Indian Bael) leaves were collected from the northern part of India (Lucknow, Utter Pradesh, India) close to the Integral University campus area in August 2022. The sample was authenticated (IU/PHAR-HRBD/22/02), and the sample specimen was deposited at the College of Pharmacy, Integral University (Lucknow, India). The leaf samples were shed, dried, powdered, and volatile metabolites were isolated using the hydro-distillation (Clevenger-type) method and purified by following the preceding method [29]. The purified *A. marmelos* leaf essential oil was weighed and transferred into an air-tight vial (amber-colored) under the deep refrigeration (−20 °C) until use.

#### 4.2.2. Gas-Chromatography/Mass Spectrometry (GC-MS) Analysis of Essential Oil

The volatile components present in crude *Aegle marmelos* leaf essential oil were explored by GC/MS instrumentation investigation. The GC/MS-QP2010 equipped with an auto-sampler auto-injector (AOC-20si) instrument (Shimadzu, Tokyo, Japan) was used in this study. The conditions, such as the temperature of the column oven, injection volume, and documentation of compounds, were adopted with slight modifications to the reported analysis conditions [29]. Then, 100 mg of AMLEO was dissolved in 10 mL hexane (10 mg·mL^−1^), and 1 µL solution was injected in a split-mode (ratio, 1:10) in an RTX-5MS column where the length was 30 m, the diameter was 0.25 mm, and the film thickness of the stationary phase was 0.25 µm (Shimadzu, Japan). The system was operated at 70 eV, helium was used as a carrier gas, and the gas flow rate was set at 1.21 mL/min, with the column head pressure of 69 kPa. The injection temperature was fixed at 260.00 °C. The oven temperature was first initiated at 50 °C for 2 min; then, the temperature was increased to 180 °C (with the rate of 5 °C/min), held for 1 min, and then to 280 °C (with the rate of 15 °C/min), at which the column temperature was maintained for 15 min. The scan mode 40–650 *m/z* was used to explore the spectra of volatile compounds. The documentation of *Aegle marmelos* leaf volatile metabolites was identified using the National Institute of Standards and Technology MS-library (NIST 14). The percentage area and retention indices (RIs) of each volatile compound were calculated and observed relative to *n*-alkanes homologous series (C_7_–C_30_) and by comparing their mass spectra libraries (NIST) with the literature [61].

#### 4.2.3. Cytotoxicity Assay of Essential Oil

In the present study (KB) cells (human mouth epidermal carcinoma) were maintained in a high supplement of glucose medium with fetal bovine serum (10% FBS) and antibiotic-antifungal solution (1%) in an atmosphere of CO_2_ (5%), O_2_ (18–20%) at 37 °C in a CO_2_ incubator (Heal-force, Shanghai, China) and every two days they were sub-cultured. The KB cell line was performed preceding the earlier method [62] with some modifications. The cell suspension (200 μL) was seeded into a 96-well plate (2 × 10^4^ cells/well) and, for approximately 24 h, it was allowed to grow. An appropriate *Aegle marmelos* oil sample concentration was added and incubated for 24 h at 37 °C. Plates were removed from the CO_2_ incubator, and MTT reagent (3-(4,5-dimethylthiazol-2-yl)-2,5-diphenyl-2H-tetrazolium bromide) was added following the concentration of 0.05% of total volume. The plate was further wrapped in aluminum foil and incubated for 3 h. After 3 h, MTT reagents were removed, and 100 μL DMSO was added carefully. The absorbance was measured at 570 nm using an enzyme-linked immunosorbent assay plate reader (ELISA, Biorad-PW41, (Hercules, CA, USA). Cell viability percentage was calculated using the given formula (Equation (1)), and using a linear regression equation (Y = mx + c) the IC_50_ was determined. From the viability diagram, Y = 50, and M and C values were identified.

(1) % Cell Viability=(Absorbance of Treated Cell/Absorbance of Control Cells)×100

Morphological changes of cells were studied at different concentrations (12.5–200 μg/mL) after treatment of KB with *Aegle marmelos* oil samples using fluorescence microscopy (Leica Microsystems Inc., Buffalo Grove, IL, USA) at a magnification of 100×.

#### 4.2.4. Free Radical Scavenging Activity of Essential Oil

The method of 2,2-diphenyl-1-picrylhydrazyl (DPPH) is based on the free radical (DPPH•) produced by the oxidation of DPPH with methanol to confirm by development of purple/violet color. The antioxidant compounds have a hydrogen denoting ability to change from violet to a yellow color. In the present study, free radical scavenging powers of *Aegle marmelos* oil were analyzed using the earlier DPPH assay [63], with some modifications. Concisely, a stock solution (1000 µg/mL) of samples and the standard solution was prepared. The DPPH-cation solution (0.1 mM) was prepared in methanol. Into the 5 mL test tubes, 2 mL of DPPH was added and then the standard and samples were added separately to make a final concentration of 20–100 µg/mL. The reaction mixtures were correctly mixed and incubated in the dark for 30 min at 25 °C. The absorbance was recorded at 517 nm using UV-Visible-Spectrophotometer (Double-beam Systronics India (Pvt.) Ltd., Ahmedabad, Gujarat, India). The percentage inhibition (% I) was documented as mean ± standard deviation of triplicate.

The method of 2,2′-azino-bis (3-ethylbenzothiazoline-6-sulfonic acid) (ABTS) is based on the free radical (ABTS^•+^) produced by the oxidation of ABTS with potassium persulfate, confirmed by development of bluish green color. The antioxidant compounds have a hydrogen denoting ability to change from bluish green to colourless. In the present study, free radical scavenging powers of *Aegle marmelos* oil were analyzed using the earlier ABTS assay [64], with some modifications. Consciously, 7.5 mmol/L ABTS and 2.5 mmol/L potassium persulfate were prepared in methanol. ABTS∙+ (radical cation) was created by mixing ABTS and potassium persulfate, allowing it to keep in the dark for 16 h at room temperature before use. Later, the free radical cation stock solution (ABTS∙+) was diluted with methanol (UV Absorption: 0.90 ± 0.02 at 734 nm). Similar to the DPPH assay, in the 5 mL test tube, 1.9 mL of ABTS∙+ solution and standard samples were added to make a final concentration of 20–100 µg/mL to each test tube. The absorbance was recorded after 7 min at 737 using UV-Visible-Spectrophotometer (Double-beam, Systronics India (Pvt.) Ltd., Ahmedabad, Gujarat, India). The percentage radical scavenging (%RA) was documented as mean ± standard deviation of triplicate (Equation (2)).

% RA = [(Ab (DPPH/ABTS − Ab (sample/standard)/Ab (DPPH/ABTS) × 100)](2)

A graph plotting the percentage of inhibition versus concentration was used to calculate the IC_50_ value (the essential oil concentration providing 50% inhibition) of antioxidant activity.

#### 4.2.5. Anti-*S. mutans* Activity of Essential Oil

The well-diffusion method determined the antibacterial potential of *Aegle marmelos* oil [54]. Plates containing nutrient agar (Hi-media, Mumbai, India) media were used to test *Aegle marmelos* oil effects on *Streptococcus mutans* (MTCC 389). The inoculum of the selected bacterial suspension was prepared in 10 mL nutrient broth. The suspension turbidity was adjusted to 1.5 × 10^6^ colony-forming units/mL using 0.5 McFarland. Then, 50 μL of this suspension was poured onto fresh solidified agar media plates. Four wells of 6 mm diameter were bored in the inoculated media. Each board was filled with 50 μL of various concentrations (50, 100, and 200 μg/mL) of *Aegle marmelos* oil and allowed to diffuse for 30 min, followed by incubation at 37 °C for 24 h. The inhibition of growth zones around the well was measured in mm, and means ± standard deviation were calculated. The solutions dimethyl sulfoxide (10%) and amoxicillin (10 μg/mL) were also used as a negative and positive controls, respectively. For the minimum inhibitory concentration (MIC), different concentrations (1.25, 2.5, 5, to 10 μg/mL) of AMLEO were prepared in 10% DMSO. The MIC was determined using the broth dilution CLSI method [33]. To each diluted tube, 100 µL of bacterial inoculum was seeded in nutrient broth (NB). Aliquots (100 μL) of each dilution were added in each tube and incubated at 37 °C for 24 h. The lowermost concentration that produced no visible growth was recorded as MIC. To investigate the bactericidal anti-*S. mutans* activity of *Aegle marmelos* oil, the time-kill assay of Aftab et al. was performed [33]. The cultures of *S. mutans* were incubated on the broth of nutrient agar and cultured at 37 °C for 10 h.

The suspension of *S. mutans* was centrifuged and resuspended in saline at 1.5 × 10^6^ CFU/mL. The *S. mutans* suspensions were treated with broth medium containing different concentrations of *Aegle marmelos* oil according to the MIC, mixed, and cultured at 37 °C. At the selected intervals (0, 3, 6, 12, and 24 h), samples (1/2 × MIC and 1 × MIC) were removed, diluted with saline, and re-cultured and incubation of the plates at 37 °C for 24 h in a broth medium. After that, the CFU/mL (colony forming unit/mL) was calculated, and a graph, log 10 CFU/mL versus time, was plotted to explore the time-kill assay.

#### 4.2.6. Molecular Docking Effects of Major Compound Limonene

The molecular docking study of the ligands (D-limonene) and the 3D structure of the protein molecules were performed using ADT to determine their potential binding affinities. The tool ADT (AutoDock) created the file from the conventional RCSB PDB files [65,66]. For the molecular docking study, 3NM8: tyrosinase X-ray structure of *B. megaterium* tyrosinase [67] and 3CC6: protein tyrosine kinase 2 beta (PTK2B) were selected for the prediction of antioxidant properties of limonene [68] For the anti-*S. mutans* docking study, 3OPU: C-terminal domain of Streptococcus mutans surface proteins SpaP and 3QP1: CviR ligand-binding domains bound to the native ligand C6-HSL were selected for the inhibitory effect of limonene [69,70].

To draw the 3D input files, only the PDB structure of the protein part was taken by removing additional ions, atoms, and molecules [71]. All protein targets were actively minimized by using the CHARMM software tool [72]. The 2D structures of D-limonene (CID: 440917) ligands from the PubChem database in the standard data format (SDF) were retrieved [33]. The standard 2D data format was transformed to PDB-3D using the BIOVIA discovery studio visualizer [73]. The ligands and receptors, a file of grid parameters, and the PDF file of docking parameters were produced to execute the molecular docking. The grid box size for the coordinates x, y, and z was 40 Å, with a grid spacing of 0.4200 Å, and center of the grid box in the x (28.095 Å), y (27.0005 Å), and z (8.4735 Å). Around the protein molecule, the grid frame was drawn with different grid points on the axes of x, y, and z and a maximum distance (1.00) between two successive grids to deliver sufficient space for ligand movement.

Ten (10) runs were executed for the protein and ligand. The minimum free binding energy (ΔG) and inhibition constant (Ki) were considered selective parameters to pick one of the best positions of the ligands bound to the binding cleft of the receptor [74].

### 4.3. Statistical Analysis

Statistics of the present study were explored using GraphPad Prism 9.5.0 software (GraphPad Software, Inc., San Diego, CA, USA) and are presented as mean ± standard deviation. The test sample concentration that created 50% inhibition (IC_50_ value) was evaluated using the graph by plotting the inhibition (%) versus the concentration. A significant *p*-value (* *p* < 0.05, ** *p* < 0.01, *** *p* < 0.001) was determined when the test sample was compared with a standard compound using a paired *t*-test.

## 5. Conclusions

In conclusion, the research conducted on *Aegle marmelos* leaves indicates that they possess valuable natural therapeutic properties for managing oral cancer and infections. The essential oil extracted from these leaves exhibits significant anticancer, antioxidant, and anti-cariogenic effects. Specifically, the volatile metabolites in the oil inhibit DPPH- and ABTS-induced free radicals as well as *Streptococcus mutans* bacteria growth in in vitro studies. Molecular modelling of limonene compounds present in *Aegle marmelos* leaves demonstrated theoretical inhibitory effects on proteins associated with antioxidant and antibacterial activity, which supports our experimental results indicating strong ligand-protein-binding energy interactions (ΔG) and inhibition constants (µM). Therefore, it is suggested that *Aegle marmelos* leaves can be considered an alternative source for managing human mouth epidermal carcinoma as well as bacterial infections due to their potent anticarcinoma potential against KB cells along with remarkable performance against *S.mutans*. Therefore, *A. marmelos* leaves could be considered as an alternative treatment option to conventional medicines for oral cancer and infections with their well-established traditional medicinal benefits combined with modern scientific validation through this study’s results.

## Figures and Tables

**Table 1:** Chemical composition of the *A. marmelos* leaf essential oil.

Composition	RI (Lit)	RI (Obs)	Area %
Monoterpenes			
α-Pinene	932	948	0.19
Myrcene	988	958	0.73
Limonene	972	976	63.71
*β*-Ocimene	1022	1018	1.73
cis-Sabinene hydrate	1059	1041	0.35
Linalool	1082	1082	0.45
p-Menth-2,8-dien-1-ol	1138	1140	2.29
D-Carvone	1210	1190	1.76
cis-Carveol	1209	1206	1.10
(-)-Myrtenol, TMS derivative	1221	1221	1.06
trans-2-Hydroxy-1,8-cineole	1247	1247	6.85
Menthyl acetate	1294	1304	2.83
trans-p-menth-8-ene-1,2-diol	1340	1346	0.95
Sesquiterpenes			
γ-Elemene	1430	1431	0.19
trans-β-Caryophyllene	1417	1414	1.05
α-Bisabolene	1521	1518	0.66
Globulol	1548.1	1530	0.71
Ledene alcohol	1570	1561	0.54
Caryophyllene oxide	1582	1587	2.04
Humulene-1,2-epoxide	1608	1592	0.34
Rosifoliol	1600	1598	0.48
Diterpene			
Geranyl-α-terpinene	1960	1962	0.28
Other compounds			
3-Methyl-2-(2-methyl-2-butenyl)-furan	1099	1109	0.45
Trimethyl[2-(phenylthio)ethoxy]silane	1214	1228	0.94
6-Allyl-2-cresol	1316	1316	0.40
Pentanoic acid, 2-methyl-, anhydride	1347.5	1339	2.92
(+)-3-Carene, 2-(acetylmethyl)-	1380	1384	0.29
(2E)-7-Ethoxy-3,7-dimethyl-2-octen-1-ol	1463	1431	0.24
beta-Ionone epoxide	1456	1436	0.48
10,12-Hexadecadien-1-ol	1880	1870	0.39
9,12,15-Octadecatrienoic acid, ethyl ester, (Z,Z,Z)-	2145	2201	0.25
9,12-Octadecadiynoic acid, trimethylsilyl ester	2201	2221	0.53
13-Hexyloxacyclotridec-10-en-2-one	2071.2	2325	0.58
Cholesta-3,5-diene	2580	2390	0.23
Monoterpenes (84.02%)			
Sesquiterpenes (6.01%)			
Diterpene (0.28%)			
Non-terpene compounds (8.01%)			
Total % Area (98.32%)			

RI (Lit) = Retention Index determined in literature (NIST and PubChem) and RI (Obs) = Retention Index determined in reference to a homologous series of n-alkanes (C7–C29).

**Table 2:** The molecular docking score, ki, and residues for limonene with target antioxidant and antimicrobial proteins.

Enzymes	PDB: ID	Binding Energy ΔG (kcal/mol)	Inhibition Constant (µM)	Hydrogen Bond Interaction
Tyrosinase	3NM8	−7.73	898.74	PHE A: 194, VAL A: 215, HIS A: 57, HIS A: 201, HIS A: 205
Protein tyrosine kinase 2 beta (PTK2B)	3CC6	−7.40	859.54	ARG A: 134, PRO A: 165, LEU A: 156
C-terminal domain of *S. mutans* surface protein	3OPU	−7.31	987.29	PRO A: 59, LYS A: 110
CviR ligand-binding domain bound to the native ligand C6-HSL	3QP1	−7.89	764.34	ILE A: 106, HIS A: 105, PHE A: 99, TYR A: 52, LEU A: 120, VAL A: 53

## Data Availability

The data presented in this study are available on request from the corresponding author.
